# Case-Control Study of Risk Factors Associated with Feline and Canine Chronic Kidney Disease

**DOI:** 10.4061/2010/957570

**Published:** 2010-09-20

**Authors:** Paul C. Bartlett, James W. Van Buren, Andrew D. Bartlett, Chun Zhou

**Affiliations:** ^1^College of Veterinary Medicine, Michigan State University, 171 Food Safety Building, E. Lansing, MI 48824, USA; ^2^Vector Scientific Resources, Inc., 5780 Braeburn Court, Kalamazoo, MI 49009, USA; ^3^1505 Gilcrest Ave., E. Lansing, MI 48823, USA; ^4^Hill's Pet Nutrition Pty Limited, 350 Orchard Road #18-07, Shaw House, Singapore 238868

## Abstract

An age-matched case-control study was initiated to determine the major risk factors associated with CKD in cats and dogs and to determine what clinical signs cat and dog owners observed before their veterinarian diagnosed their pet with CKD. When compared to controls, the feline cases were more likely to have had polydipsia and polyuria in the year before the owners' cats were diagnosed with CKD. In the dogs, increased water intake, increased urination, small size and a recent history of weight loss and bad breath were noticed by the dog owners before veterinary CKD diagnosis. Dog owners recognized abnormal drinking and urination behavior over half a year before their pet's veterinary diagnosis with CKD, and they recognized weight loss almost 4 months before CKD diagnosis. Bad breath was noticed 1.2 years before recognition of CKD by a veterinarian. Given that earlier CKD diagnosis should have been possible in most cases, clinical trials should proceed to measure the efficacy of early interventions.

## 1. Introduction

Chronic kidney disease (CKD) has been reported in 9.6% of cats examined at Purdue's teaching hospital, and recently we reported kidney diseases affecting 7.6% (361/4,731) of the feline caseload at four U.S. university teaching hospitals [1, 2]. Rates are higher in older cats, with Krawiec and Gelberg [3] reporting a CKD rate of 30% in cats over 15 years old and Bartlett [2] reporting kidney disease among 28% (97/348) of all examined cats over 12 years of age. The prevalence of CKD in dogs has been reported as 5.8% of the veterinary caseload [2], and is manifested primarily as a geriatric disease.

The clinical course of CKD is progressive and irreversible, with eventual uremia and azotaemia [4]. Clinical signs reportedly associated with decreased kidney function include loss of appetite, vomiting, loss of body weight, and increase in water consumption and urine production (polydipsia/polyuria) [5, 6]. Some animals with CKD die within a few months of diagnosis, while others remain stable for years [7]. A CKD staging system for dogs and cats has been established by the International Renal Interest Society (IRIS), and the stage of renal disease is believed to be strongly associated with patient survival time [8]. 

Dietary modification has been the principle intervention attempted to retard the progression of CKD [1, 4, 9–12]. Survival times are reportedly increased if dietary modification for CKD is initiated early in the clinical course of the disease before the onset of azotemia and chronic renal failure [5, 13]. Lees [13] states that in order to realize the maximum benefit of dietary modification, veterinarians (and presumably clients) must become more astute in detecting early renal disease in their patients. 

In this regard, one of our objectives was to determine the risk factors and/or early signs of CKD that were noticed by the owners before their pet received a veterinary diagnosis of CKD. If early detection by owners and confirmation by primary care veterinarians are possible, then the next step would be to conduct blinded clinical trials to determine the efficacy of CKD intervention. Another objective was to identify risk factors for CKD that might elucidate possible causal factors and enable primary prevention.

## 2. Materials and Methods

Because age is such an important CKD determinant, cases and controls were matched on the basis of age and secondarily on year of veterinary treatment. All cases and age-matched controls were taken from the 2006–2008 caseload of Michigan State University's (MSU) Veterinary Teaching hospital (VTH). Cases were selected if they met all of the following three criteria: (1) diagnosis of CKD as evidenced by one or more of the SNOMED codes for acute-on-chronic renal failure, renal failure syndrome, or chronic renal failure syndrome; (2) serum creatinine of IRIS Stage 2 or higher (1.6 mg/dL or higher in cats or 1.4 mg/dL or higher in dogs); (3) urine-specific gravity of <1.030 or trace (or greater) of protein in urine. Application of the above case definition resulted in the identification of 168 feline cases and 236 canine cases.

**Selection of Controls**

After exclusion of research animals or animals presented for spay/neuter clinics, feline and canine patients from 2006–2008 were eligible for selection as controls. Controls were matched to cases on the basis of species (feline or canine), animal age, and by the time of treatment at the hospital. Within species, age, and date groups, controls were selected as having the nearest patient number to the case's patient number. (Case numbers were sequentially assigned when each animal is first presented for treatment.) One control and a backup control were selected for each case, but the backup control was only contacted if the first control did not respond to the survey. It was confirmed from the hospital's medical records that the controls had no record of any renal disease. Additionally, two questions on the survey requested information regarding the existence of any renal disease, and potential controls were excluded from the study if there was any indication that the control cat or dog had ever had any renal disease.

**Data Collection and Processing**

The feline risk factor survey was written and reviewed by the authors and a specialist in companion animal veterinary internal medicine. Some survey directions for the cases and controls were worded slightly differently to acknowledge the fact that the control cats had never been diagnosed with CKD and to ensure that case owners understood that we were asking only about observations before their pet's first veterinarian-confirmed diagnosis with CKD. For the controls, pet owners were queried about observations before the CKD onset date in their matched case so that both case and control owners were being asked to recall events from the same number of months in the past. The owners of feline cases and controls were mailed a survey and recontacted in about 1 month with a 2nd mailed survey. About one month after the 2nd mailing, nonrespondents were called on the telephone and asked to return the survey and offered an additional mailed survey. Messages were left on answering machines if the pet owner did not answer the phone.

After completion of the feline data in the summer of 2009, the risk factor survey was modified for administration to canine cases and controls, which began in the fall of 2009. Like the feline survey, the canine survey asked about the existence of various risk factors (observations, conditions, and diseases) occurring before the time of diagnosis in the case, but also asked the case owners to estimate how many years and months before the CKD diagnosis the risk factor was first noticed. Dog owners were asked to estimate the number of pounds of body weight which their pet was over or under its ideal weight at 4, 3, 2, and 1 years before the time of CKD diagnosis in the case.

**Statistical Analysis**

Data was entered into a spreadsheet and proof read with a second person. The data was then moved to SAS for analysis. (SAS 9.1.3 Cary, NC. USA) Categorical variables were analyzed by SAS proc logistic for a matched-pair case-control study, producing an odds ratio with 95% confidence intervals. Odds ratios were considered statistically significant at *P* < .05 if the confidence interval did not contain 1.0. For continuous level variables, cases and controls were compared to each other using a paired t-test for matched case-control studies. Multivariable analysis on the feline data was attempted to adjust for confounding among variables found to be statistically significant as lone predictors of CKD.

Each of the canine dichotomous variables was evaluated independently as described above for its association with CKD. Variables found statistically significant at *P* < .05 were then evaluated in all possible two-variable models. Because of the large number of significant variables and the limited sample size, not all variables could be evaluated in a single multivariable model. One model was created to determine the best predictor(s) of excessive water intake and urination. A second model was created to determine the best predictor(s) of weight loss. A third model evaluated miscellaneous variables related to bad breath and lethargy. The best predictors from these three models of different biologic systems were then evaluated in a combined model. Variables eliminated during the multivariable modeling phase were tested with the final combined model to confirm that their inclusion did not provide a significant contribution.

## 3. Results

Our survey response rate was 55% (92 completed pairs/168 selected pairs) for the cats and 51% (120/236) for the dogs. The cats with CKD average 15 years of age (SD = 5.0), and the dogs averaged 9.9 years of age (SD = 3.9).

**Feline Analysis**

The odds ratios for dichotomous variables are shown in Table 1 and the paired *t*-test results for continuous variables are shown in Table 2. Early recognized clinical signs, diseases, and other observations that were significant at *P* < .05 included excessive thirst, frequent urination, and urinating large volumes. Factors that approached statistical significance included hyperthyroidism, lower urinary tract disease, frequent vomiting, refusal to eat, decreased appetite, unexplained weight loss, percent of diet from dry food, and percent of diet from hunting. For the feline data, no two-variable models were significant due to the barely significant risk factors and the small number of case-control pairs available for analysis.

**Canine Analysis**

A variable for the difference in weight (in units of 10 Kg) between the case and its matched control was found to be a significant predictor of CKD at *P* = .0004, with an odds ratio of 1.5 (1.2–1.9) for every decrease in weight of 10 kg. This means that each decrease in animal weight of 10 kg was associated with a 50% increase in CKD. Eleven other risk factors were found to be associated with CKD (Table 3). Weight contributed significantly to all three of the biologic systems models (Table 4) as well as the final combined model (Table 5). The analysis of the continuous canine variables is shown in Table 6.

Cases were reportedly overweight by 1.5, 1.2, 1.2, and .64 kg at 4, 3, 2 and 1 year before CKD onset, respectively. The difference in kg over or under ideal weight between the case and matched control was always *P* > .5 by paired *t*-test, although this analysis was hampered by missing values for up to 50% of the matched pairs. At 4 years before CKD onset in the case, the controls were reportedly over their ideal weight by 0.27,  0.14,  0.32, and 0.64 more kg than were the cases. The current body weight, or body weight about 1 month before death, was reported to be 28 kg for the controls and only 20 kg for the cases (Table 6), indicating that the cases were more likely to be smaller breed dogs than were the controls.

## 4. Discussion

The feline and canine surveys repeatedly stressed that we were only asking about observations made before the veterinary diagnosis of CKD in the cases or, for controls, the onset date we provided for their matched case. As such, recall bias should have been minimized in that the owners of both cases and controls were asked to remember events from an equal distance into the past. We acknowledge that the evaluation of so many risk factors involves multiple comparisons that must certainly affect the validity of the generated confidence intervals, however this was a hypothesis-generating study for which no causal associations should be implied or assumed.

### 4.1. Feline CKD

When compared to their matched controls, the feline cases were more likely to have had polydipsia and polyuria in the years before their cat was diagnosed with CKD. This suggests that earlier diagnosis and intervention may have been possible if these pet owners had been taught to recognize the early signs of CKD. Given the progressive nature of the disease and the demonstrated correlation between clinical chemistry and survival times, it is reasonable to hypothesize that interventions would be most effective if initiated as early as possible in the clinical course of the disease [12, 14]. Other signs such as weight loss and decreased appetite approached statistical significance, and would require a larger study to indicate if they could also be used for early recognition of feline CKD.

Our desire for a specific diagnosis (inclusion criteria 2 and 3) reduced the study sample size such that, when coupled with a return rate of 55%, we only had 92 completed pairs for analysis. Another unexpected challenge to statistical power was that many of the risk factors were quite rare in the study population. Therefore, there are several risk factors that have reasonably high odds ratios, but failed to reach statistical significance in that the OR confidence interval included 1.0. 

The association with hyperthyroidism had a high odds ratio (4.5). Although both hyperthyroidism and CKD are strongly associated with age, the cases and controls were matched for age, so age cannot explain this observed association. It is possible that cats for which clinical pathology results were obtained were more likely to have been diagnosed with both hyperthyroidism and CKD, which are both very common in older cats. 

There was a nonsignificant finding that the percent of diet from hunting might be positively associated with CKD, and further investigation may be warranted. This question was asked because infectious or toxicologic agents associated with hunting, such as hantavirus in rodents, may contribute to renal destruction as a hidden cause of CKD.

### 4.2. Canine CKD

Most significant variables related to polydipsia, polyuria, recent weight loss, small body size, and periodontal disease. Controls were somewhat more likely to get a greater percentage of their diet from dry dog food, but this was most likely a result of their averaging about 8 kg more than their matched cases. The two-variable models shown in Table 4 provide information regarding which variables provide unique predictive information and which variables are duplicative. For example, Ur_more and Ur_large are each significant predictors by themselves, but when included together they were found to provide essentially the same predictive information for CKD.

Model 1 in Table 6 shows that the variable for excessive thirst was the best predictor relating to excessive urination and drinking. When combined with animal weight, no other variable could significantly add to the prediction. Model 2 regarding eating behavior and weight loss showed that three variables (a history of weight loss, refusal to eat and decreased appetite) all had independent predictive ability for CKD. Model 3 showed that lethargy and bad breath were independently predictive of CKD.

The combined final model (Table 5) included the most significant effects from all three component models, and included body weight, excessive thirst, bad breath, history of weight loss, and refusal to eat. These results suggest that education of owners for the early signs of CKD should focus on recognition of old, small-breed dogs that have bad breath, a history of weight loss and/or decreased appetite, which begin to drink an excessive amount of water and urinate large volumes. We found that these observations were recognized by their owners many months before the dogs were diagnosed with CKD by their veterinarian.

Polzin et al. [15] reports that 15% of dogs 10 years of age or older and 33% of cats 15 years of age or older have structural and functional changes of the kidney. In our age-matched study, the average age of the cats (15 yrs) and the dogs (9.9 yrs) supports the designation of CKD as a geriatric disease of both cats and dogs. Because our study used age-matched controls, we were not able to study the effect of age. 

A small body size was an extremely important predictor of CKD in dogs, such that future studies should consider matching on body size (or breed) as well as age. We found that our cases averaged 8 kg less weight than controls. This was the current weight or weight 2 months before death for dogs that were deceased. We also found that a lower body weight was a significant predictor of CKD in all of our multivariable models (Tables 5 and 6). Very little of this decreased weight in cases was due to the early signs of CKD. 

If CKD can be recognized sufficiently early in its clinical course, there are several prospects for intervention which presumably would be the most effective if introduced as early as possible during the clinical course of the disease. Dietary modification has been reported to retard the development of CKD in dogs and cats [1, 5, 10, 15–21]. Diets used typically have reduced protein, phosphorus, sodium content [15]. Different dietary rations may be optimal during the various stages of disease, as determined on the basis of clinical signs and laboratory data [22]. Systemic hypertension commonly accompanies CKD in dogs and may also need to be controlled to prevent more rapid disease progression [23]. 

In cats, it has been recommended that serum phosphate and parathyroid hormone be monitored, and phosphate-restricted dietary management and intestinal phosphate binders be used to retard the progression of CKD and accompanying hyperparathyroidism [24]. Angiotensin-converting enzyme inhibitor (ACEI) benazepril in cats with CKD produced a significant reduction in proteinuria, and may hold promise as a supplement to nutritional intervention [25]. In one of the few blinded clinical trials, dogs fed a diet designed for renal disease had a slower decline in renal function and a decreased mortality when compared to controls fed an adult maintenance diet [21].

High rates of gingivitis and periodontal disease have been reported in humans with chronic kidney disease [26–30]. In dogs, periodontal disease has been related to pathologic lesions in the kidney [31, 32]. These findings are consistent with the more frequent reports of bad breath and dental plaque that we found in our CKD cases. Bad breath and excessive dental plaque may be one of the most easily recognizable early warning signs of incipient CKD.

## Figures and Tables

**Table 1:** Feline-matched case-control odds ratios for dichotomous owner-reported risk factors for CKD, with lower (LC) and upper (UC) 95% confidence limits.

Risk factor description	Odds ratio	LC	UC
Being female	.91	.49	1.7
Being neutered	—	—	—
Excessive thirst or drinking more than a usual amount of water^1^	4.6	2.0	10
Urinating more frequently than usual^1^	4.0	1.5	11
Urinating larger volume than usual^1^	7.5	1.7	33
Urinating in the wrong place (not in the litter box or outside)^1^	.47	.19	1.1
Unexplained weight loss^1^	1.6	.90	3.0
Visible loss of muscle/strength ^1^	1.0	.42	2.4
Unexplained weight gain^1^	—	—	—
Decreased appetite^1^	1.4	.76	2.7
Refusal to eat for > than one day^1^	1.8	.74	4.7
Vomiting (more frequent, not just hairballs)^1^	1.1	.64	2.1
Lethargic (excessive sleeping, fatigue, low-energy)^1^	1.2	.60	2.5
Constipation^1^	.88	.32	2.4
Excessive gas (flatulence)^1^	—	—	—
Dental problems^1^	1.0	.43	2.3
Gum problems (oral ulcers, etc.)^1^	.83	.25	2.7
Excessive plaque or tartar on teeth^1^	.90	.37	2.2
Bad breath^1^	.90	.37	2.2
Excessive skin or coat odor^1^	.57	.11	4.0
Abnormal or poor hair coat^1^	.79	.36	1.7
Pale mucous membranes (eyes, mouth, etc.)^1^	.33	.067	1.7
Eye problems or changes^1^	.55	.20	1.5
Mobility problems (stiffness or limping)^1^	1.2	.51	2.9
Loss of vision^1^	.20	.023	1.7
Loss of hearing^1^	.60	.14	2.5
Never had teeth professionally cleaned	.96	.55	1.7
Water always available^2^	—	—	—
Having water refill only once per day^2^	.52	.25	1.1
Cat was outside at least some of the time^2^	.84	.43	1.6
Sometimes exposed to other cats^2^	.71	.32	1.6
Some exposure to house mice^2^	.66	.37	1.2
Lower urinary tract disease (including infection, stones, cystitis, plugged, etc.)^2^	1.5	.72	3.1
Neurological disease^2^	.67	.11	4.0
Seizures^2^	1.0	.14	7.1
Liver problems^2^	.20	.023	1.7
Dental problems^2^	.87	.41	1.8
Behavioral problems^2^	.63	.20	1.9
Neoplasm (cancer)^2^	.43	.11	1.7
Eye disease^2^	1.3	.34	4.7
Glaucoma^2^	—	—	—
Ear infections or other ear problems^2^	.57	.17	2.0
Frequent vomiting (not just hairballs)^2^	.81	.39	1.7
Gastrointestinal problems ^2^	1.2	.39	3.5
Parasitic problems^2^	2.5	.49	13
Reproductive problems^2^	—	—	—
Obesity^2^	.93	.44	2.0
Diabetes^2^	2.0	.37	11
Hyperthyroidism^2^	4.5	.97	21
Musculoskeletal (muscles, bones)^2^	.33	.035	3.2
Hip dysplasia^2^	—	—	—
Dermatologic (skin diseases)^2^	1.2	.37	3.9
Cardiovascular diseases (heart disease)^2^	.75	.26	2.2
Osteodystrophy^2^	—	—	—

^1^Observations in the one year before the CKD diagnosis in the case.

^2^Observations in all years before the CKD diagnosis in the case.

**Table 2:** Feline-Analysis of Continuous variables regarding observations before the diagnosis of CKD in the Case, analyzed by matched *t*-test.

Lifetime description of diet before diagnosis of ckd in the case	Case mean	Control mean	Paired *P* value
Lifetime % of diet-dry food^1^	72	73	.84
Lifetime % of diet-wet food^1^	24	24	.84
Lifetime % of diet-table scraps^1^	2.2	1.5	.45
Lifetime % of diet-moist (bag or pouch)^1^	1.2	2.2	.48
Lifetime % of diet-hunting^1^	.86	.72	.78
Dry food percent^2,3^	.95	1.0	.10
Wet food percent^2,3^	.54	.63	.24
Table scraps percent^2,3^	.21	.25	.67
Moist food percent^2,3^	.072	.053	.52
Hunted food percent^2,3^	.47	.18	.08
Mature diet percent^2,3^	.47	.42	.29
% of years outside^2,3^	8.12	13	.14
Water available^2^ (1 = sometimes, 2 = usually, 3 = always)	39	38	.74
Avg number of other cats contacted^2^	1.6	1.8	.40

^1^Asked of owner regarding the cat's lifetime experience.

^2^Tabulated from the owner's account of each year of the cat's life.

^3^This percent of lifetime diet was calculated from the owner's assessment of the cat's diet during each year of its life.

**Table 3:** Canine-matched case-control odds ratios for dichotomous owner-reported risk factors for CKD, with lower (LC) and upper (UC) 95% confidence limits.

Risk factor description	Odds ratio	LC	UC	Yrs^1^ noticed before Dx (SD)
Being female	.968	.59	1.6	
Being neutered	.55	.20	1.5	
Excessive thirst or drinking more than a usual amount of water: (x_thrst)	4.4	2.3	8.4	.74 (2.1)
Urinating more frequently than usual: (Ur_more)	4.7	2.2	10	.71 (2.2)
Urinating larger volume than usual: (Ur_large)	3.8	1.6	9.4	.51 (2.0)
Urinating in the wrong place: (Ur_acc)	2.7	1.4	5.4	.60 (1.8)
Unexplained weight loss: (Wgtloss)	9.5	3.4	27	.38 (1.8)
Visible loss of muscle/strength	2.0	.90	4.5	
Unexplained weight gain	3.0	.81	11	
Decreased appetite: (Dec_ap)	5.1	2.5	10	.43 (1.5)
Refusal to eat for > than one day: (Refusal)	11.7	3.6	38	.22 (1.2)
Vomiting (more frequently than other dogs): (Vomit)	3.5	1.4	8.7	.45 (2.0)
Lethargic (excessive sleeping, fatigue, low-energy): (Leth)	2.3	1.2	4.2	.16 (.45)
Constipation	1.7	.40	7.0	
Excessive gas (flatulence)	1.6	.52	4.9	
Dental problems	1.6	.64	3.9	
Gum problems (oral ulcers, etc.)	4.0	.45	36	
Excessive plaque or tartar on teeth: (Plaque)	2.6	1.2	5.5	.80 (2.5)
Bad breath: (Breath)	2.9	1.4	6.0	1.2 (2.9)
Excessive skin or coat odor	2.7	.71	10	
Abnormal or poor hair coat	.70	.30	1.6	
Pale mucous membranes (eyes, mouth, etc.)	1.3	.34	4.7	
Eye problems or changes	1.1	.41	3.2	
Mobility problems (stiffness or limping)	.62	.33	1.1	
Loss of vision	1.6	.73	3.5	
Loss of hearing	1.1	.41	3.2	
Teeth professionally cleaned at least yearly	1.23	.73	2.1	
Water always available	—	—	—	
Outside more than just for walks	.56	.26	1.2	
Frequently exposed to dogs outside the household	.65	.32	1.3	
Sometimes or frequently exposed to mice	1.1	.53	2.2	
Inappropriate urination, blood in urine, painful urination, infection, stones, cystitis, plugged, and so forth.	.90	.37	2.2	
Neurological disease	.5	.13	2.0	
Seizures	1.4	.54	3.8	
Liver problems	4.5	.97	21	
Dental problems	2.0	.81	5.0	
Behavioral problems	3.0	.61	15	
Neoplasm (cancer)	.47	.19	1.1	
Eye disease	1.1	.41	3.2	
Glaucoma	2.0	.18	22	
Ear infections or other ear problems	.63	.33	1.2	
Gastrointestinal problems	.64	.25	1.6	
Parasitic problems	.46	.16	1.3	
Reproductive problems	—	—	—	
Obesity	1.6	.77	3.3	
Diabetes	2.0	.18	22	
Hyperthyroidism	1.0	.20	5.0	
Musculoskeletal (muscles, bones)	.69	.30	1.6	
Hip dysplasia	.33	.07	1.7	
Dermatologic (skin diseases)	.79	.40	1.6	
Cardiovascular diseases (heart disease)	4.0	.45	36	
Osteodystrophy	—	—	—	

^1^Number of years before veterinary diagnosis when this disease was diagnosed or observation first noticed in the CKD case. Shown only for statistically significant risk factors.

**Table 4:** Biologic systems models regarding urinary symptoms (Model 1), weight loss (Model 2) and miscellaneous (Model 3)—adjusted for dog weight^1,2,3^.

	OR	LC	UC		OR	LC	UC
Model 1							
X_thrst	5.5	2.5	12	Wtkg10	1.7	1.3	2.2

Model 2							
Wgt_Loss	5.8	1.6	21	Refusal	8.3	1.6	43
Dec_ap	3.2	1.1	8.9	WtKg10	1.6	1.2	2.2

Model 3							
Leth	2.5	1.2	5.1	Breath	2.6	1.1	6.0
WtKg10	1.4	1.1	1.8				

^1^OR = Odds ratio estimate with the lower (LC) and upper (UC) 95% confidence interval.

^2^Variables are defined in Table 3.

^3^The following variables were evaluated.

Model 1: WtKg10 x_thrst, *Ur_more Ur_large Ur_acc,*

Model 2: WtKg10 Wgtloss Dec_ap Refusal *vomit,*

Model 3: WtKg10 Leth *Plaque* Breath,

Nonsignificant variables (underlined) were removed.

**Table 5:** Combined model^1^.

Variable	OR	LC	UC	Sig
Dec_ap	4.5	1.7	12	.003
Breath	3.6	1.1	12	.034
Wgtloss	9.4	2.1	43	.004
X_thrst	5.6	2.0	16	.001
KgWt10	1.6	1.2	2.3	.004

^1^Variables are defined in Table 3

Max-rescaled *R* Square = 0.6824.

**Table 6:** Canine-analysis of continuous variables regarding observations before the diagnosis of CKD in the case, analyzed by matched *t*-test.

Lifetime description of diet before diagnosis of CKD in the case	Case mean	Control mean	Paired *P* value
Age in years	9.9	10	.061
Lifetime % of diet-dry food	75	82	.042
Lifetime % of diet-wet food	14	8.4	.047
Lifetime % of diet-table scraps	10	9.2	.74
Lifetime % of diet-moist (bag or pouch)	1.7	.76	.22
Food for all ages (last 2 yrs of life)^1^	51	49	.66
Mature diet (last 2 yrs of life)^1^	24	26	.57
Diet for overweight (last 2 yrs of life)^1^	6.8	6.3	.85
Weight in kg^1^	20	28	.00

^1^Percent of diet of this type during the last 2 years of life.
